# Developing a novel model for predicting overall survival in late-onset colon adenocarcinoma patients based on LODDS: a study based on the SEER database and external validation

**DOI:** 10.1007/s12672-025-01849-0

**Published:** 2025-01-29

**Authors:** Chen Chen, Heng-Bo Xia, Wei-Wei Yuan, Meng-Ci Zhou, Xue Zhang, A.-Man Xu

**Affiliations:** 1 Department of General Surgery, Anhui Public Health Clinical Center, Hefei, 230000 China; 2 Department of General Surgery, The First Affiliated Hospital of Anhui Medical University, Hefei, 230000 China; 3 Department of Interventional Radiology, Affiliated Hospital of Xuzhou Medical University, Xuzhou, 221000 China

**Keywords:** LODDS, Late-onset colon adenocarcinoma, Prognostic model, Overall survival, Cause-specific survival

## Abstract

**Aim:**

To construct a predictive model based on the LODDS stage established for patients with late-onset colon adenocarcinoma to enhance survival stratification.

**Methods:**

Late-onset colon adenocarcinoma data were obtained from the public database. After determining the optimal LODDS truncation value for the training set via X-tile software, we created a new staging system by integrating the T stage and M stage. Nomograms of the prognostic model were created after Cox analyses identified independent risk factors for overall survival (OS) and cause-specific survival (CSS) and were validated internally and externally. The efficacy of the nomograms was assessed by calibration, time-dependent area under the curve (AUC) and decision curve analysis (DCA). Finally, the prognoses of the patients were compared by plotting survival curves on the basis of risk scores.

**Results:**

A total of 103,291 and 100 patients with late-onset colon adenocarcinoma (50–80 years old) were screened from the Surveillance, Epidemiology, and End Results (SEER) and The Cancer Genome Atlas (TCGA) databases, respectively. Cox regression analysis revealed independent risk factors for OS and CSS, including age, gender, race, size, LODDS stage, PLN stage, LNR stage, and TNM stage. A comparison of the four models constructed on the basis of different stages revealed that the model constructed with the LODDS stage had the minimum AIC (Akaike information criterion), maximum C-index (concordance index) and time-dependent AUC. Nomograms based on the LODDS stage were constructed and successfully validated for accuracy and clinical utility.

**Conclusion:**

For patients with late-onset colon adenocarcinoma, LODDS may achieve optimal predictive performance. Furthermore, compared to the 8th edition of the AJCC classification system, the nomogram based on LODDS stage may demonstrate superior survival prediction capabilities.

**Supplementary Information:**

The online version contains supplementary material available at 10.1007/s12672-025-01849-0.

## Introduction

Colorectal cancer is a common malignant tumor of the gastrointestinal tract and is the third most common cancer in the world after breast and lung cancers with an age-adjusted incidence rate of 26.7 per 100,000 people and a mortality rate of 13.7 per 100,000 people, with colon adenocarcinomas accounting for more than 90% of the cases [1, 2]. Despite the increasing abundance of technologies and means for the diagnosis and treatment of colon cancer, its cancer-related mortality rate is still the second highest in the world [3]. Most colon cancers occur in individuals over the age of 50, with the average age at diagnosis being 68 years for men, whereas the average age at diagnosis is 72 years for women [4]. Although younger patients with colon cancer have more aggressive disease, they still have the opportunity to receive better comprehensive treatment [5, 6]. The prognosis for young patients may be better in some cases when their physical condition and treatment tolerance are taken into account. Since older patients are often diagnosed at an advanced stage of the disease and the treatment they receive is often inadequate, this may account for the low survival rate of older patients with colon cancer [7]. Given that elderly individuals are usually associated with multiple chronic diseases and physiological changes, the clinical manifestations and therapeutic strategies of colon cancer for this age cohort may be unique, and the study of the characteristics and prognosis of patients with late-onset colon cancer may better address the health care needs of this population, optimize the allocation of healthcare resources, and increase the quality of life of elderly patients with colon cancer [8]. Elderly patients often receive inadequate lymph node assessment compared with younger colon cancer patients. Improved lymph node assessment leads to more accurate pathologic staging in elderly individuals [9]. Despite the fact that the TNM staging system is currently the most commonly used system for prognostic assessment and course determination in patients with CC, it has some hidden shortcomings that limit its application [10–12]. In the AJCC staging system, the N classification is not sufficient for accurately assessing the extent of lymph node metastasis [13]. Therefore, there is an urgent need for new metrics to develop better prognostic nomograms to predict OS and CSS in late-onset colon adenocarcinoma patients.

Recently, several studies have shown the significance of the logarithmic probability of positive lymph nodes (LODDS) in predicting the prognosis of multiple types of tumors [14–16]. Previous studies have shown that the LODDS is a reliable prognostic indicator for colon cancer, regardless of lymph node status and number, and can identify patients with the same prognosis well. It shows higher accuracy in predicting prognosis than the N, PLN (positive lymph node) and LNR (lymph node ratio) staging systems [17]. In a recent study, the LODDS was proposed as a novel prognostic indicator for colon and noncolon cancer [18–21]. Nevertheless, the prognostic value of creating a new prognostic classification for patients with late-onset colon adenocarcinoma by incorporating T stage and M stage is still unknown.

Hence, in this research, we intended to create a LODDS stage for patients with late-onset colon adenocarcinoma, and on the basis of this, we developed a predictive model that has been shown to better predict the prognosis of late-onset colon adenocarcinoma patients by comparing it with various staging systems, such as the TNM staging system.

## Methods

### Data resources

**Fig. 1:**
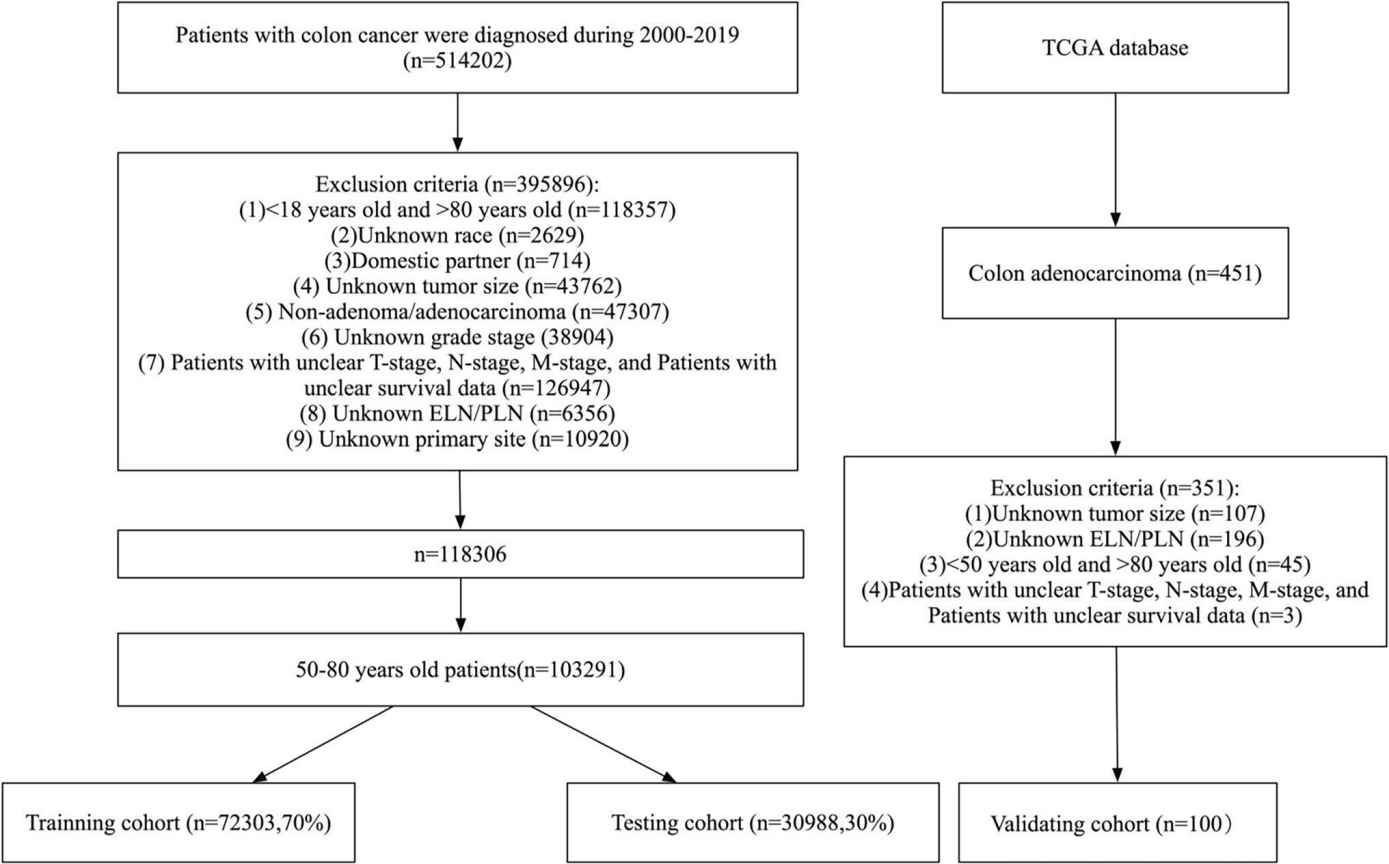
Flowchart of data collection and grouping for patients with late-onset colon adenocarcinoma

Data on CC patients were obtained from the two most representative tumor databases, SEER and TCGA. The study was exempt from ethical approval because the data were retrieved from public databases [22]. We gathered data from the SEER database on CC patients who underwent surgery between 2000 and 2019. The exclusion criteria for extracted data included the following: (1) patients younger than 50 years and older than 80 years of age at diagnosis; (2) unknown race; (3) domestic partner; (4) unknown tumor size; (5) nonadenoma/adenocarcinoma; (6) unknown grade stage; (7) patients with unknown T, N, or M stage and unclear survival data; (8) unknown ELN/PLN; and (9) unknown primary site. All patients were randomised into a training group [72,303 (70%)] or a testing group [30,988 (30%)] using the “Caret package” in the R (version 4.3.1) software. Using the same exclusion criteria, using the same exclusion criteria, we obtained a total of 100 data points from the TCGA database for validation as an external validation group (Fig. 1).

### Measurement of variables

Race was categorized into three subsets: white, black, and others. The “Others” covered AK Native, Asian, American Indian, and Pacific Islanders. Marital status was classified into six subdivisions: married, divorced, separated, single, widowed and unknown. The primary site was grouped into the right side of the colon (transverse colon), the left side of the colon and the large intestine (NOS). The sizes were grouped into nine clusters: d < 1 cm, 1 ≤ d < 2 cm, 2 ≤ d < 3 cm, 3 ≤ d < 4 cm, 4 ≤ d < 5 cm, 5 ≤ d < 6 cm, 6 ≤ d < 7, 7 ≤ d < 8 cm, and ≥ 8 cm. The T stage contains six subsets, Tis, T1, T2, T3, T4a and T4b. The N stage included four subsets: N0, N1/N1C, N2a and N2b. The M stage was organized into two subsets: M0 and M1. The new staging system was determined with reference to the 8th edition of the AJCC staging system. The LNR was defined as the ratio of PLNs to ELNs. LODDS was obtained from the following equation: log[(PLN + 0.5)/(ELN−PLN + 0.5)]. To avoid errors when dividing by zero, 0.5 is added to both the numerator and denominator. The primary endpoints are OS, and the secondary endpoints are CSS and are represented in the SEER database as “COD to Field Record” and “SEER Specific Cause of Death Classification”, respectively.

### Best thresholds for variables

The best thresholds for LODDS, LNR, and PLN were calculated from minimum p-values and maximum chi-square values via X-tile (version 3.6.1) software. The resulting best thresholds for LODDS were categorized as follows: LODDS1 (− 2.26 to − 1.26), LODDS2 (− 1.26 to − 0.45), and LODDS3 (− 0.45 to 1.86). The best thresholds for the LNR were as follows: LNR1 (0–0.04), LNR2 (0.04–0.24), and LNR3 (0.24–1). The best thresholds of PLN were categorized as PLN1 (= 0), PLN2 (1–2), and PLN3 (≥ 3). Then, we compared the predictive ability of lymph node staging methods, including LODDS, PLN, LNR, and N, using the time-dependent AUC approach.

### Constructing a LODDS stage classification

**Fig. 2:**
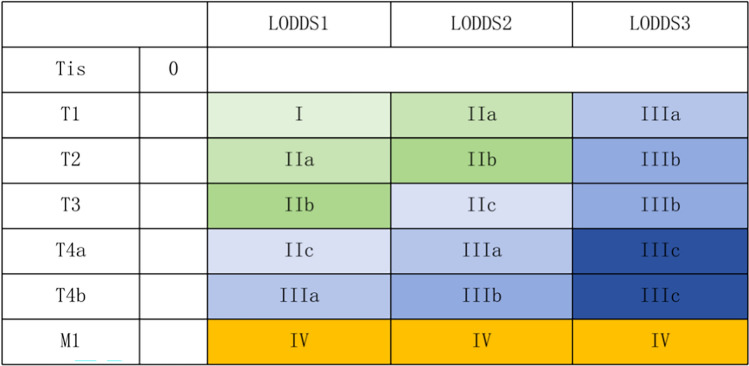
A novel LODDS stage classification

For the training group, the LODDS were classified into LODDS1, LODDS2, and LODDS3 according to the optimal thresholds of LODDS. We explored a potential staging framework comprising 19 sub-stages ((Tis, T1–4bLODDS1–3M0–1) by combining the LODDS, T and M stages, inspired by the 8th edition of the AJCC classification. And the 19 sub-stages were processed into 9 groups: stage 0 (Tis), I (T1LODDS1M0), IIa (T2LODDS1M0, T1LODDS2M0), IIb (T3LODDS1M0, T2LODDS2M0), IIC (T4aLODDS1M0, T3LODDS2M0), IIIa (T4bLODDS1M0, T4aLODDS2M0, T1LODDS3M0), IIIb (T4bLODDS2M0, T2-3LODDS3M0), IIIc(T4a-4bLODDS3M0) and IV (M1) (Fig. 2). The same categorization was applied to the PLN stage and LNR stage.

### Creation, assessment, validation and application of the LODDS stage model

In the training set, we screened for independent influences on OS and CSS via Cox univariate and multivariate analyses to construct the model. The discrimination ability and model fit of the LODDS stage model, PLN stage model, LNR stage model and TNM stage model were further compared. The predictive efficiency of these models was compared via the AIC, C-index and AUC via the “risk regression” R package. Subsequently, between LODDS stage and TNM stage, we further compared the clinical prognostic predictive ability of the two models for the corresponding sub-stages.

### Constructing and validating nomograms

Nomograms drawn by integrating the variables in the screening model that predicted the OS and CSS of the training group with the highest accuracy (via the “rms” R package). The validity of the nomograms was assessed via the C-index, the time-dependent AUC, and calibration plots in the training and testing sets. The clinical performance of the nomograms was assessed via the R software package “ggDCA”.

### Survival risk classifier via the nomogram

To further verify the performance of the nomograms, all the patients were categorized into high- and low-risk groups on the basis of the total score computed by the “survival” R package. Differences in survival between the two risk groups in terms of OS and CSS were assessed via the Kaplan‒Meier method.

### Statistical analysis

Statistical analyses of categorical variables are expressed as percentages, and comparisons between groups were made via the χ^2^ test. Normally distributed continuous variables are expressed as the means ± SDs, and independent samples t tests were used for comparisons between groups. Nonnormally distributed continuous measurements are expressed as medians (interquartile spacings), and comparisons between groups were made via the Mann‒Whitney U nonparametric test, with P < 0.05 indicating statistical significance. All the statistical analyses were performed via R (version 4.3.1) software, and all the graphs were drawn via R software and GraphPad Prism (version 4.3.0).

## Results

### Patient characteristics and survival

A total of 103,291 and 100 patients with late-onset colon adenocarcinoma (50–80 years old) were screened from the Surveillance, Epidemiology, and End Results (SEER) and The Cancer Genome Atlas (TCGA) databases, respectively. In the SEER database, 72,303 (70%) of the included patients were assigned to the training set, and the remaining 30,988 (30%) patients were assigned to the internal validation cohort. Table 1 shows the demographic and clinicopathologic characteristics of the patients in the training and testing groups, which were not significantly different (p > 0.05).

**Table 1:** Clinical and pathological characteristics of late-onset colon adenocarcinoma patients in the two cohorts

Characteristics	Overall n = 103,291	Training set n = 72,303	Testing set n = 30,988	P value
Age	68.00 [61.00, 74.00]	68.00 [60.00, 74.00]	68.00 [60.00, 74.01]	0.300
LN-examined	16.00 [12.00, 22.00]	16.00 [12.00, 22.00]	16.00 [12.00, 22.01]	0.847
LN-positive	0.00 [0.00, 1.00]	0.00 [0.00, 1.00]	0.00 [0.00, 1.01]	0.811
LNR	0.00 [0.00, 0.08]	0.00 [0.00, 0.08]	0.00 [0.00, 0.09]	0.850
LODDS	− 1.36 [− 1.57, − 0.88]	− 1.36 [− 1.57, − 0.88]	− 1.36 [− 1.57, − 0.89]	0.739
Survival-months	74.00 [43.00, 116.00]	75.00 [43.00, 116.00]	75.00 [43.00, 116.01]	0.106
Gender (%)				
Male	53,961 (52.2)	37,757 (52.2)	37,757 (52.3)	0.840
Female	49,330 (47.8)	34,546 (47.8)	34,546 (47.9)	
Race (%)				
Black	12,971 (12.6)	9031 (12.5)	9031 (12.6)	0.423
White	81,245 (78.7)	56,950 (78.8)	56,950 (78.9)	
Others	9075 (8.8)	6322 (8.7)	6322 (8.8)	
Marital-status(%)				
Married	60,439 (58.5)	42,389 (58.6)	42,389 (58.7)	0.227
Divorced	10,458 (10.1)	7366 (10.2)	7366 (10.3)	
Separated	1039 (1.0)	725 (1.0)	725 (1.1)	
Single	13,555 (13.1)	9365 (13.0)	9365 (13.1)	
Widowed	13,498 (13.1)	9440 (13.1)	9440 (13.2)	
Unknown	4302 (4.2)	3018 (4.2)	3018 (4.3)	
Primary-site (%)				
Right colon	62,546 (60.6)	43,874 (60.7)	43,874 (60.8)	0.250
Left colon	40,280 (39.0)	28,115 (38.9)	28,115 (38.10)	
Large intestine, NOS	465 (0.5)	314 (0.4)	314 (0.5)	
Grade (%)				
Well	9816 (9.5)	6933 (9.6)	6933 (9.7)	0.285
Moderately	76,258 (73.8)	53,261 (73.7)	53,261 (73.8)	
Poorly	14,917 (14.4)	10,499 (14.5)	10,499 (14.6)	
Undifferentiated	2300 (2.2)	1610 (2.2)	1610 (2.3)	
Stage (%)				
0	1367 (1.3)	963 (1.3)	963 (1.4)	0.959
I	27,029 (26.2)	18,952 (26.2)	18,952 (26.3)	
IIa	32,489 (31.5)	22,731 (31.4)	22,731 (31.5)	
IIb	1509 (1.5)	1054 (1.5)	1054 (1.6)	
IIc	1116 (1.1)	792 (1.1)	792 (1.2)	
IIIa	4340 (4.2)	3034 (4.2)	3034 (4.3)	
IIIb	20,789 (20.1)	14,580 (20.2)	14,580 (20.3)	
IIIc	3864 (3.7)	2704 (3.7)	2704 (3.8)	
IV	10,788 (10.4)	7493 (10.4)	7493 (10.5)	
T-stage (%)				
Tis	1367 (1.3)	963 (1.3)	963 (1.4)	0.965
T1	13,410 (13.0)	9412 (13.0)	9412 (13.1)	
T2	18,794 (18.2)	13,148 (18.2)	13,148 (18.3)	
T3	60,084 (58.2)	42,043 (58.1)	42,043 (58.2)	
T4a	6146 (6.0)	4281 (5.9)	4281 (5.10)	
T4b	3490 (3.4)	2456 (3.4)	2456 (3.5)	
N-stage (%)				
N0	65,954 (63.9)	46,164 (63.8)	46,164 (63.9)	0.915
N1	27,054 (26.2)	18,951 (26.2)	18,951 (26.3)	
N1c	906 (0.9)	626 (0.9)	626 (0.10)	
N2a	4843 (4.7)	3406 (4.7)	3406 (4.8)	
N2b	4534 (4.4)	3156 (4.4)	3156 (4.5)	
M-stage (%)				
M0	92,503 (89.6)	64,810 (89.6)	64,810 (89.7)	0.198
M1	10,788 (10.4)	7493 (10.4)	7493 (10.5)	
Size (%)				
< 1	5109 (4.9)	3618 (5.0)	3618 (5.1)	0.162
1–2	9323 (9.0)	6442 (8.9)	6442 (8.10)	
2–3	15,555 (15.1)	11,014 (15.2)	11,014 (15.3)	
3–4	19,502 (18.9)	13,595 (18.8)	13,595 (18.9)	
4–5	18,171 (17.6)	12,707 (17.6)	12,707 (17.7)	
5–6	13,319 (12.9)	9298 (12.9)	9298 (12.10)	
6–7	8672 (8.4)	6061 (8.4)	6061 (8.5)	
7–8	5417 (5.2)	3818 (5.3)	3818 (5.4)	
≥ 8	8223 (8.0)	5750 (8.0)	5750 (8.1)	

LN: lymph nodes; LNR: lymph node ratio; LODDS: log odds of positive lymph nodes

### Univariate and multivariate Cox analyses for OS and CSS

**Fig. 3:**
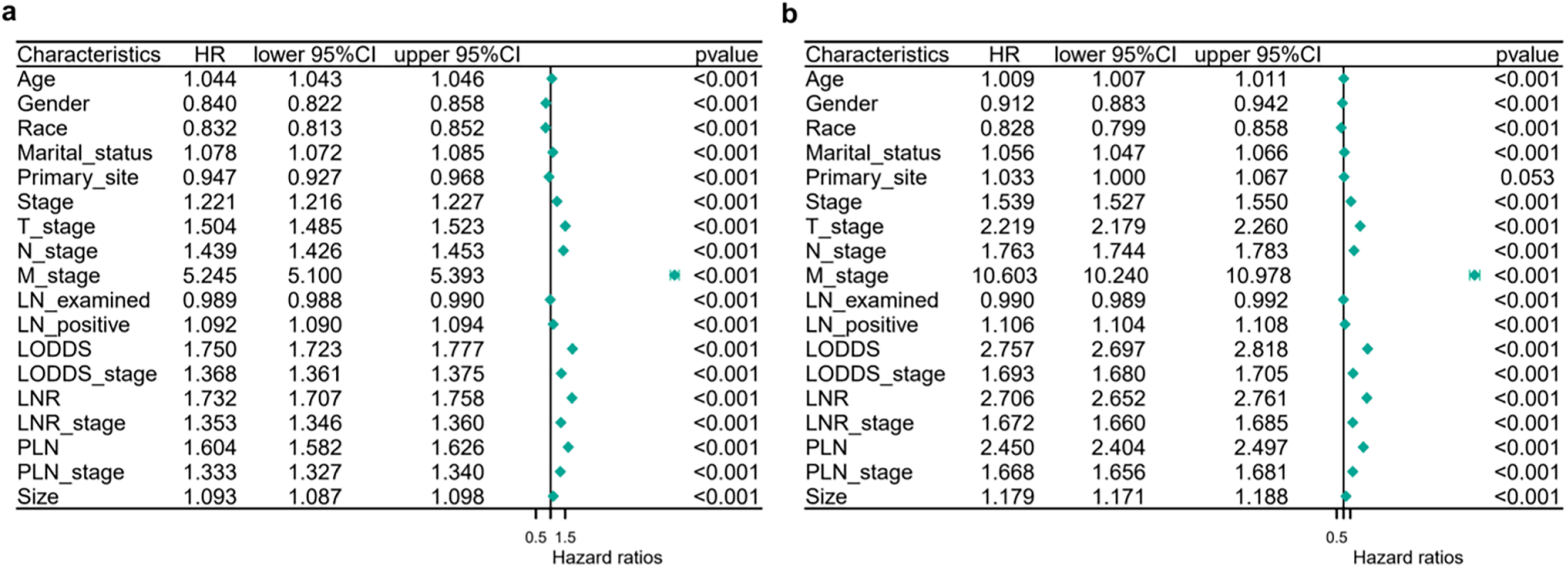
Univariate Cox regression analyses for predicting OS (**a**) and CSS (**b**) in the training cohort

We analysed 18 potential prognostic indicators via univariate Cox analysis. Figure 3 shows the results of the univariate Cox regression analysis for the training group in detail. The most significant risk factors for OS (a) and CSS (b) were age, gender, race, size, marital status, stage, T stage, N stage, M stage, LN-examined, LN-positive, LODDS, LODDS stage, LNR, LNR stage, PLN, and PLN stage. After multivariate analysis, we further generated a prognostic model that included different risk indicators. As shown in Tables 2 and 3, indicators, including age, gender, race, size, TNM stage, PLN stage, LNR stage and LODDS stage, were independent risk factors for OS and CSS.

**Table 2:** Multivariate Cox regression analyses for predicting overall survival in the training cohort

Characteristics	TNM-stage	P-value	PLN-stage	P-value	LNR-stage	P-value	LODDS-stage	P-value
	HR(95%CI)		HR(95%CI)		HR(95%CI)		HR(95%CI)	
Age	1.05 (1.05–1.05)	< 0.001	1.05 (1.05–1.06)	< 0.001	1.05 (1.05–1.06)	< 0.001	1.05 (1.05–1.05)	< 0.001
Gender	0.80 (0.78–0.82)	< 0.001	0.80 (0.79–0.82)	< 0.001	0.81 (0.80–0.83)	< 0.001	0.81 (0.80–0.83)	< 0.001
Race	0.80 (0.78–0.82)	< 0.001	0.80 (0.78–0.82)	< 0.001	0.80 (0.78–0.82)	< 0.001	0.80 (0.79–0.82)	< 0.001
Size	1.04 (1.03–1.04)	< 0.001	1.01 (1.00–1.01)	0.011	1.01 (1.00–1.01)	0.003	1.01 (1.01–1.02)	< 0.001
Primary-site	1.00 (0.98–1.02)	0.872	1.00 (0.97–1.02)	0.674	0.97 (0.95–1.00)	0.017	0.96 (0.94–0.98)	< 0.001
TNM-stage	1.23 (1.23–1.24)	< 0.001	/	/	/	/	/	/
PLN-stage	/	/	1.36 (1.36–1.37)	< 0.001	/	/	/	/
LNR-stage	/	/	/	/	1.38 (1.37–1.39)	< 0.001	/	/
LODDS-stage	/	/	/	/	/	/	1.39 (1.39–1.40)	< 0.001

LNR: lymph node ratio; PLN: positive lymph nodes; LODDS: log odds of positive lymph nodes; HR: hazard ratio; CI: confidence interval

**Table 3:** Multivariate Cox regression analyses for predicting cause-specific survival in the training cohort

Characteristics	TNM-stage	P-value	PLN-stage	P-value	LNR-stage	P-value	LODDS-stage	P-value
	HR(95%CI)		HR(95%CI)		HR(95%CI)		HR(95%CI)	
Age	1.02 (1.02–1.03)	< 0.001	1.03 (1.02–1.03)	< 0.001	1.03 (1.02–1.03)	< 0.001	1.02 (1.02–1.03)	< 0.001
Gender	0.91 (0.88–0.94)	< 0.001	0.91 (0.89–0.95)	< 0.001	0.93 (0.90–0.96)	< 0.001	0.93 (0.90–0.96)	< 0.001
Race	0.84 (0.81–0.87)	< 0.001	0.84 (0.81–0.87)	< 0.001	0.85 (0.82–0.88)	< 0.001	0.85 (0.82–0.88)	< 0.001
Size	1.07 (1.06–1.08)	< 0.001	1.04 (1.03–1.05)	< 0.001	1.04 (1.03–1.05)	< 0.001	1.05 (1.04–1.06)	< 0.001
Primary-site	0.97 (0.94–1.01)	0.094	0.97 (0.94–1.00)	0.067	0.94 (0.91–0.97)	< 0.001	0.93 (0.90–0.96)	< 0.001
TNM-stage	1.54 (1.52–1.55)	< 0.001	/	/	/	/	/	/
PLN-stage	/	/	1.68 (1.66–1.69)	< 0.001	/	/	/	/
LNR-stage	/	/	/	/	1.68 (1.67–1.69)	< 0.001	/	/
LODDS-stage	/	/	/	/	/	/	1.70 (1.69–1.71)	< 0.001

LNR: lymph node ratio; PLN: positive lymph nodes; LODDS: log odds of positive lymph nodes; HR: hazard ratio; CI: confidence interval

### Comparison of the four different models

Comparison of the four lymph node stages in the training group showed that the LODDS was a better predictor (Figure S1). Then, a comparison of the four different staging models in the training group is presented in Table 4. The C-index of the LODDS stage model was greater than that of the TNM stage model, PLN stage model and LNR stage model. Additionally, the LODDS stage model has the smallest AIC and higher time-dependent AUC values at 1, 3, 5, and 10 years than the other three models do. In addition, a comparison of the clinical prognostic predictive ability of LODDS stage model and TNM stage model showed that the predictive ability of the model of LODDS stage was better than that of TNM stage in all sub-stages (Figure S2). In summary, the LODDS stage model is much more effective in predicting OS and CSS and may be a reliable predictor.

**Table 4:** Comparison of the TNM stage model, PLN stage model, LNR stage model, and LODDS stage model

	Different models	AIC	C-index	1-year	3-yearr	5-yearr	10-yearr
OS	TNM-stage	692143.2	0.694	0.746	0.745	0.736	0.726
	PLN-stage	689100.9	0.708	0.758	0.763	0.752	0.741
	LNR-stage	688397.8	0.711	0.762	0.766	0.756	0.743
	LODDS-stage	688080.9	0.713	0.764	0.768	0.758	0.745
CSS	TNM-stage	299209.2	0.793	0.829	0.840	0.834	0.816
	PLN-stage	296781.1	0.799	0.832	0.847	0.840	0.825
	LNR-stage	296362.9	0.800	0.834	0.849	0.843	0.826
	LODDS-stage	296285.2	0.801	0.836	0.850	0.843	0.827

AIC: Akaike information criterion; C-index: concordance index; PLN: positive lymph node; LNR: lymph node ratio; LODDS: log odds of positive lymph nodes; OS: overall survival; CSS: cause-specific survival

### Constructing and validating nomograms

**Fig. 4:**
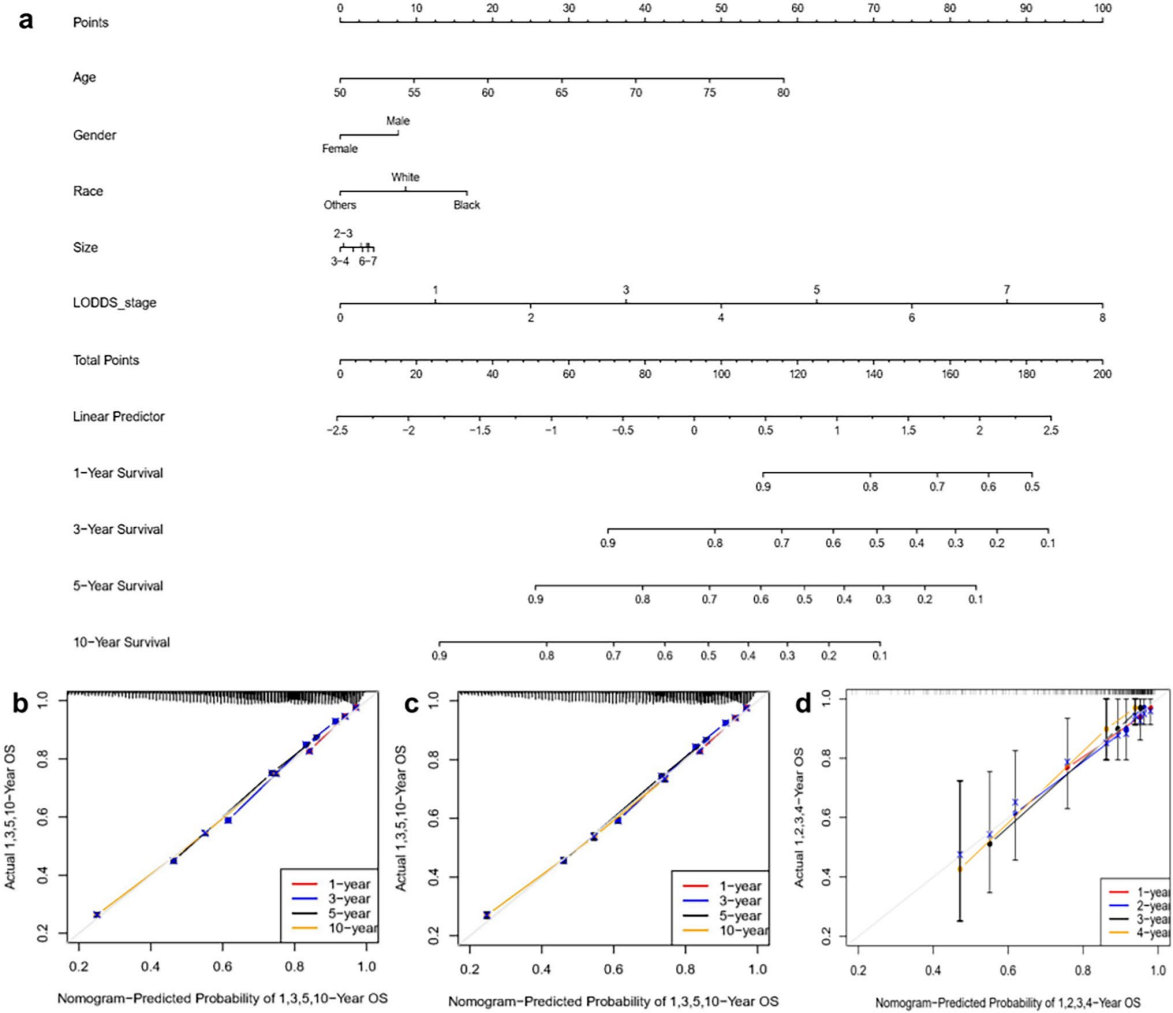
Nomogram for the OS of patients with late-onset colon adenocarcinoma. **a** Predictions of the 1, 3, 5 and 10 years OS rates via the nomogram. Calibration plots for 1, 3, 5 and 10 years in training (**b**), internal validation (**c**) and calibration plots for 1, 2, 3 and 4 years in external validation (**d**)

**Fig. 5:**
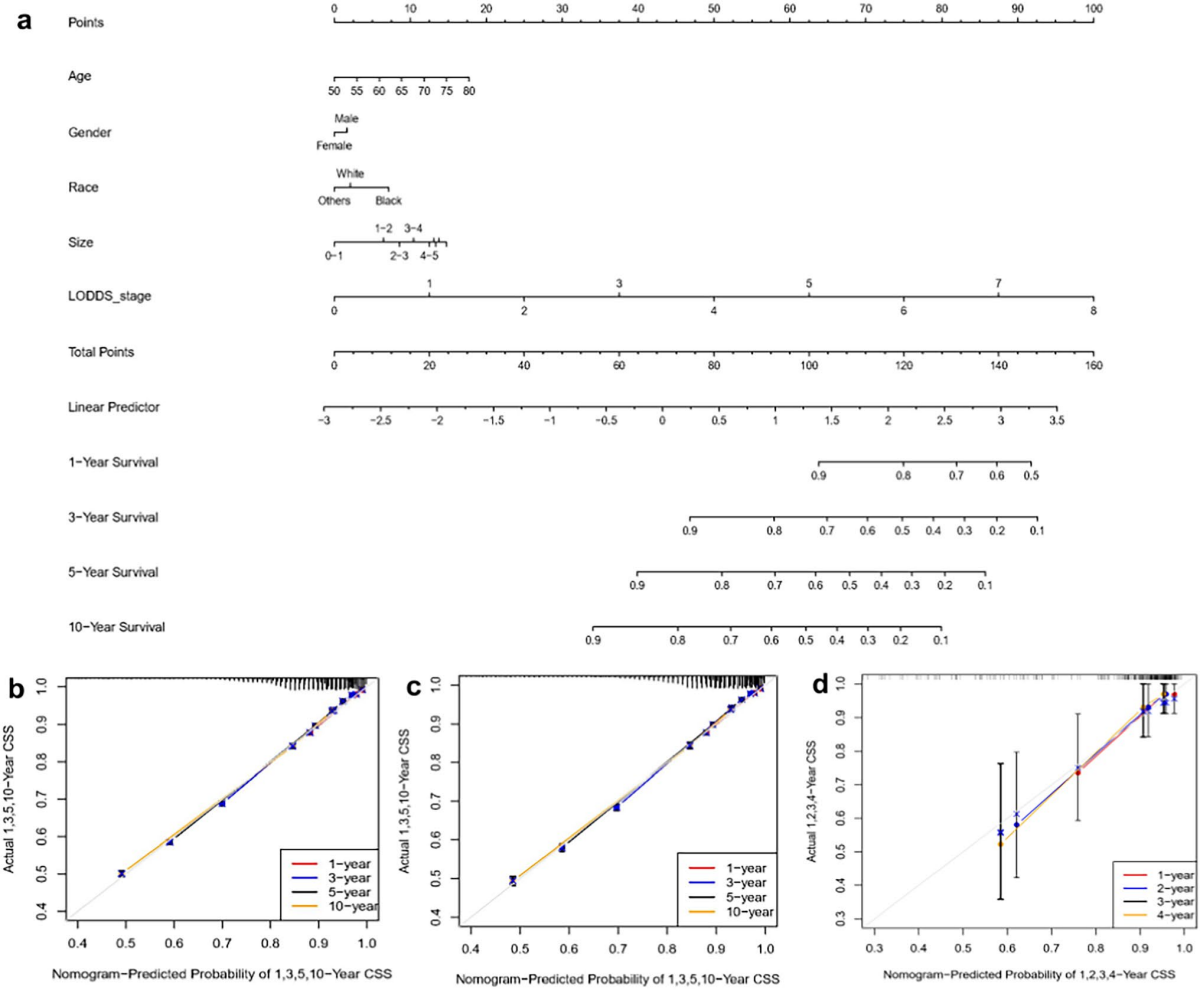
Nomogram for the CSS of patients with late-onset colon adenocarcinoma. **a** Predictions of 1, 3, 5 and 10 years CSS via the nomogram. Calibration plots for 1, 3, 5 and 10 years in training (**b**), internal validation **c** and calibration plots for 1, 2, 3 and 4 years in external validation **d**

**Fig. 6:**
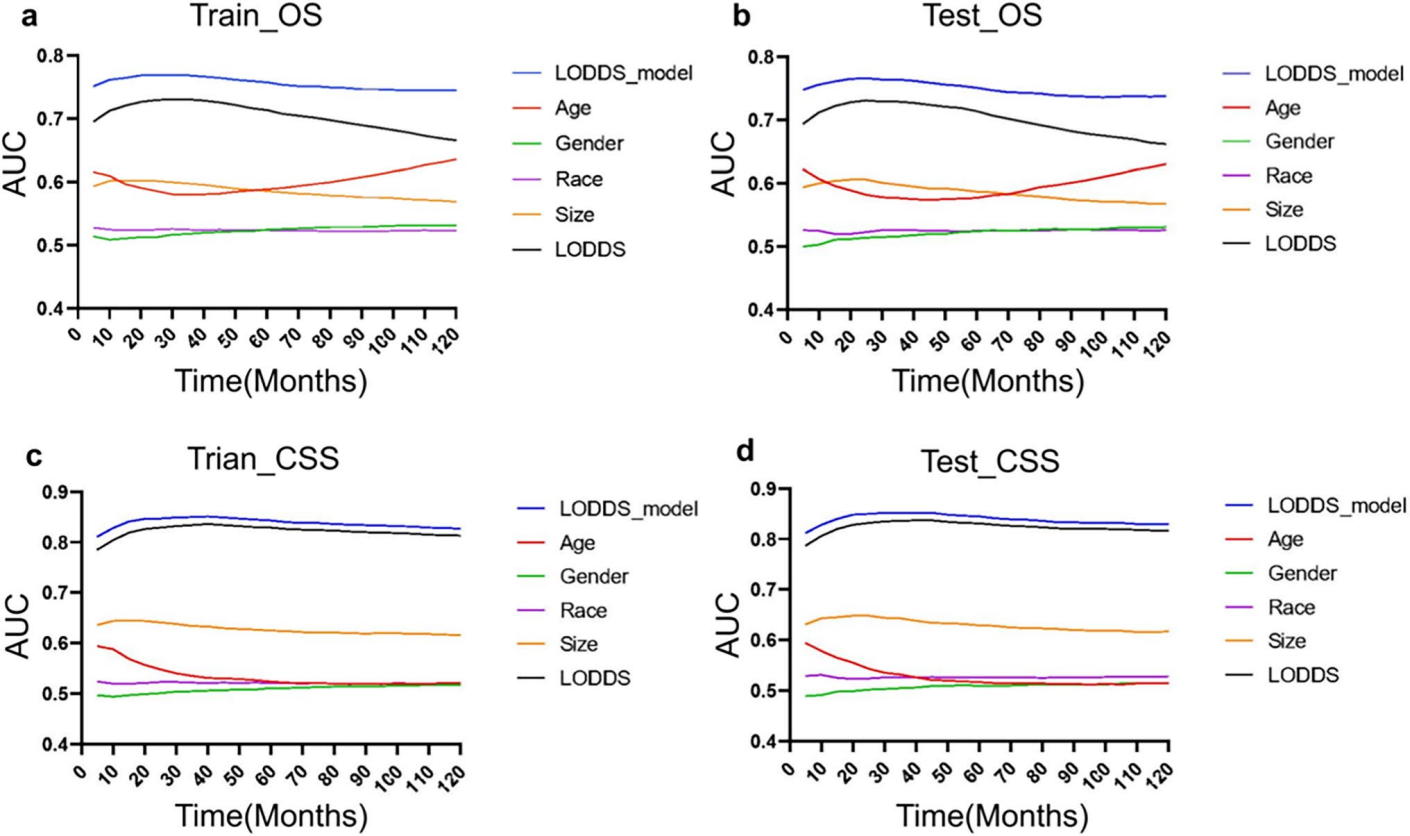
Comparison of the time‐dependent AUC of the LODDS stage model with a single characteristic for OS in the training (**a**) and testing (**b**) cohorts. Comparison of the time‐dependent AUC of the LODDS stage model with a single characteristic for CSS in the training (**c**) and testing (**d**) cohorts

**Fig. 7:**
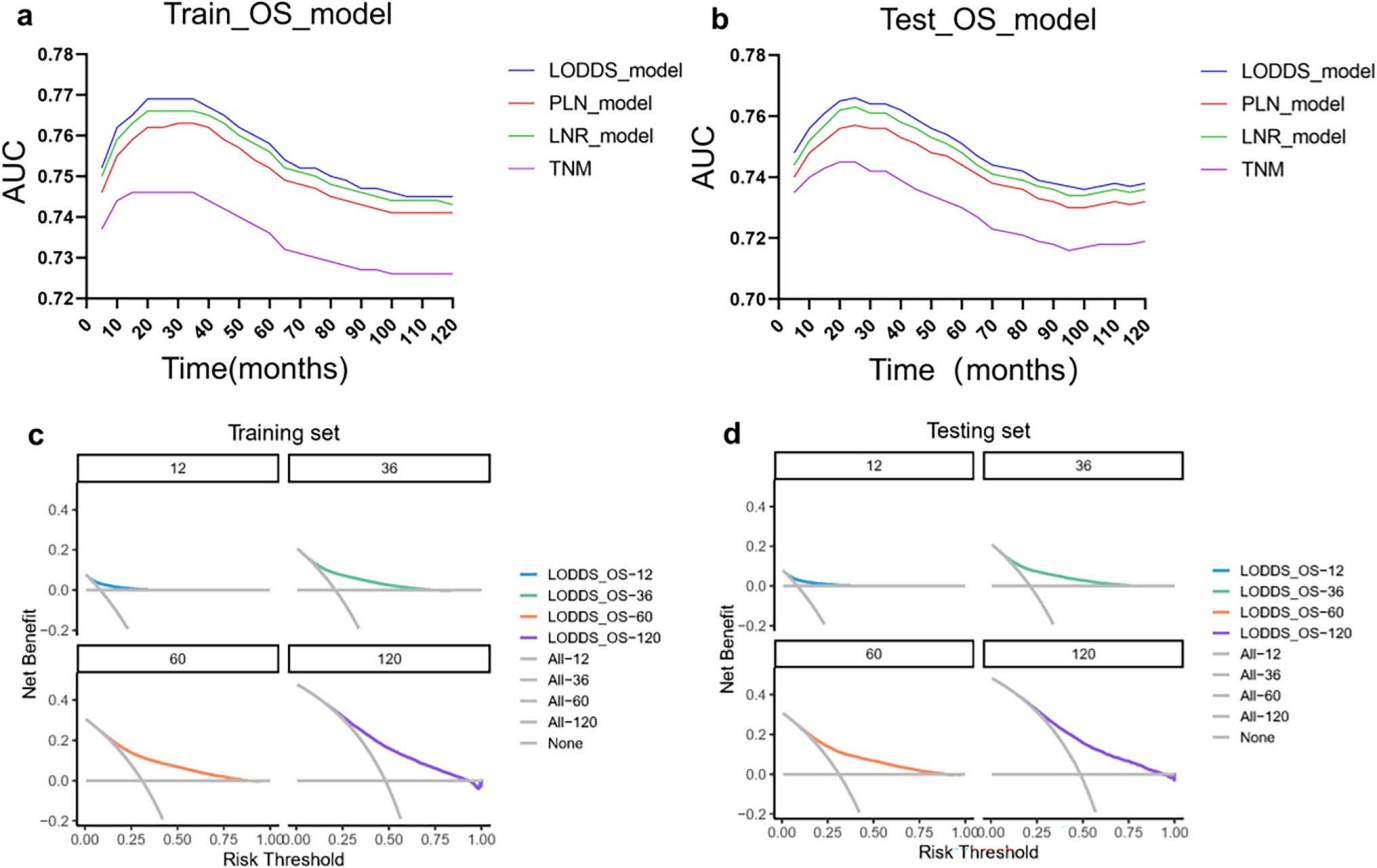
Evaluation of the nomogram for the OS of patients with late-onset colon adenocarcinoma with time-dependent AUC and DCA. The time‐dependent AUC in the training (**a**) and testing (**b**) cohorts. Decision curves for predicting 1, 3, 5 and 10 years OS in the training (**c**) and testing (**d**) cohorts

**Fig. 8:**
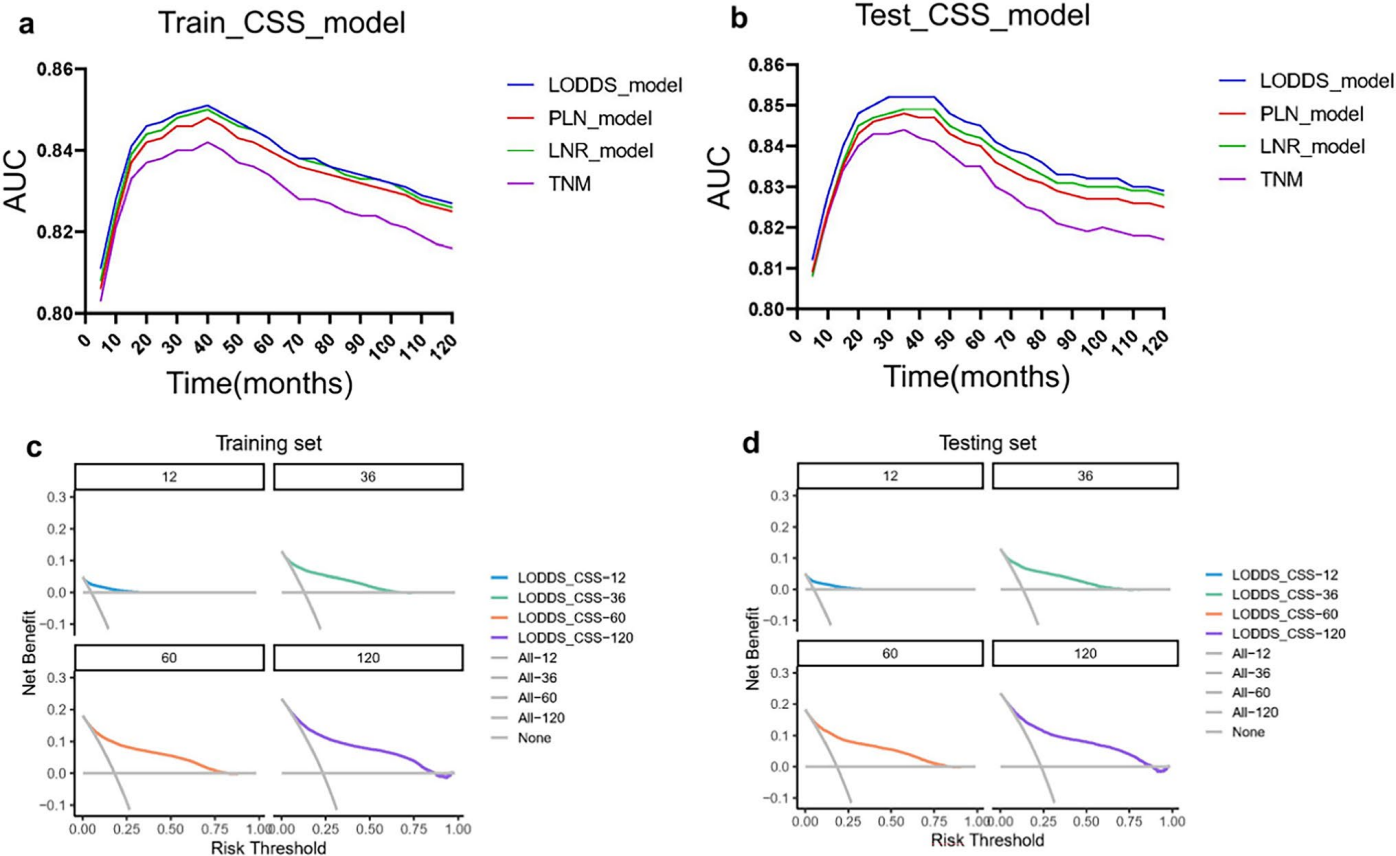
Evaluation of the nomogram for predicting the CSS of patients with late-onset colon adenocarcinoma with time-dependent AUC and DCA. The time‐dependent AUC in the training (**a**) and testing (**b**) cohorts. Decision curves for predicting 1, 3, 5- and 10 years CSS in the training (**c**) and testing (**d**) cohorts

**Fig. 9:**
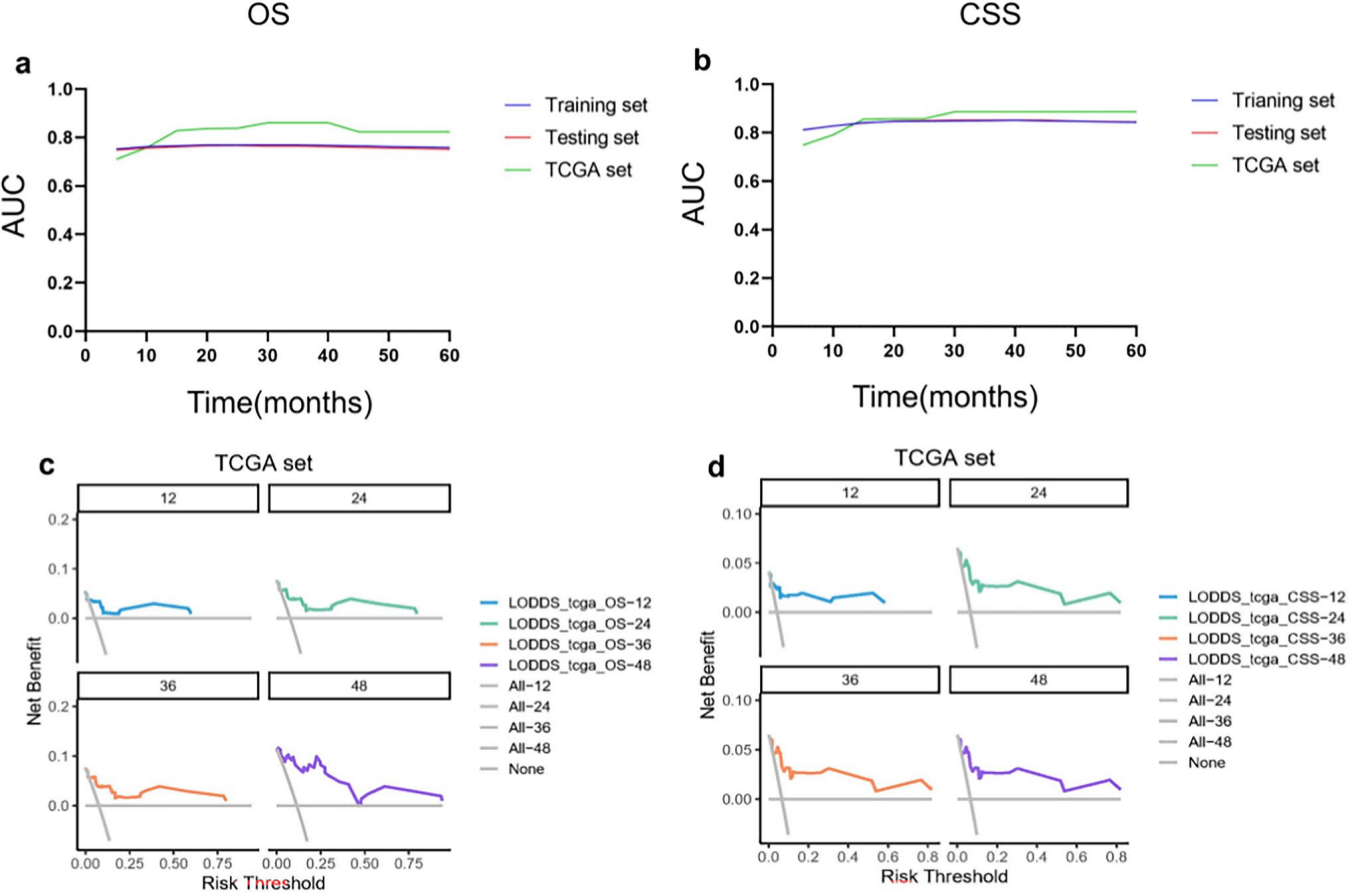
Evaluation of the nomogram for late-onset colon adenocarcinoma patients with time-dependent AUC and DCA. The time-dependent AUC for OS (**a**) and CSS (**b**) in the three cohorts. Decision curves for predicting 1, 2, 3 and 4 years OS (**c**) and CSS (**d**) in the TCGA cohort

In this study, prognostic prediction nomograms were created on the basis of five risk factors (age, gender, race, size, and LODDS stage) (Figs. 4a, 5a). The calibration plots for the three sets of data are shown in Figs. 4b–d and 5b–d, indicating that the predictive observations for OS and CSS are consistent with the practical observations. The predictive value of the LODDS stage model outperformed that of a single characteristic based on time-dependent AUC curves for OS in both the training group (Fig. 6a) and testing group (Fig. 6b) and outperformed that of a single characteristic for CSS in the training group (Fig. 6c) and testing group (Fig. 6d). In addition, the predictive value of the LODDS stage model also outperformed those of the other three models (Figs. 7a, b, 8a, b). In clinical research, decision curve analysis (DCA) has many advantages in that it can incorporate patient or decision-maker preferences into the analysis and is increasingly being used to evaluate the performance of diagnostic tests and/or predictive models [23]. Our results suggested that the LODDS stage model has better clinical application value (Figs. 7c, d, 8c, d). Because the TCGA database has relatively little survival data beyond 5 years, we only verified the survival status within 5 years. The time-dependent AUC and DCA results also demonstrated the reliability and clinical utility of the LODDS stage model in prognostic prediction for patients from the TCGA database. (Fig. 9).

### Survival risk stratification based on the predictive model

**Fig. 10:**
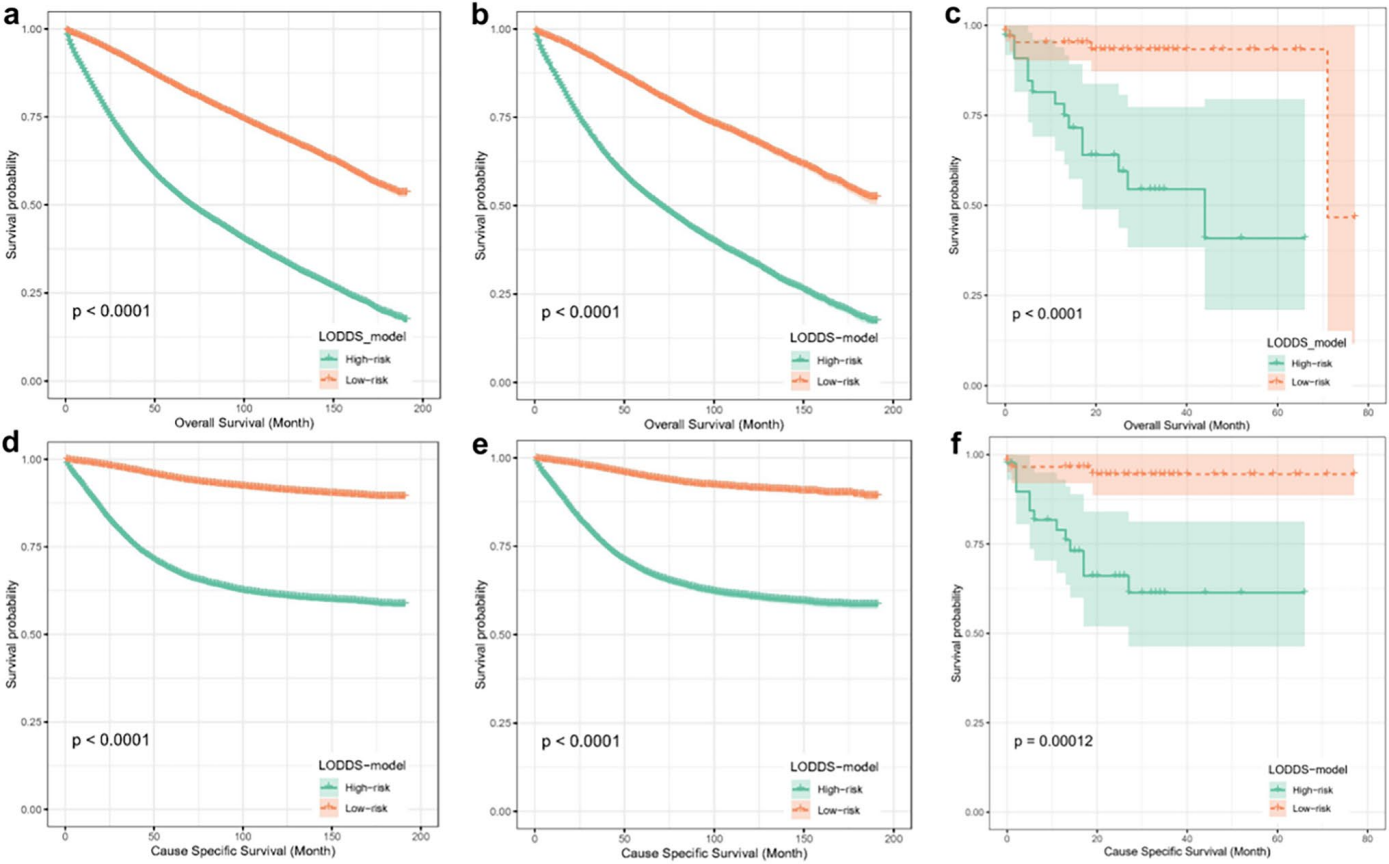
K–M analyses for late-onset colon adenocarcinoma patients classified by nomograms. K‒M curves of OS in the training cohort (**a**), testing cohort (**b**) and TCGA cohort (**c**). K‒M curves of CSS in the training cohort (**d**), testing cohort (**e**) and TCGA (**f**) cohort

To further validate the performance of the prediction model, we categorized all patients into two groups, high-risk and low-risk, using the total scores computed from the OS and CSS histograms and performed survival analysis. The Kaplan‒Meier curves revealed significantly different survival results among the three cohorts (training and testing sets from the SEER database and external validation from the TCGA database) in terms of OS and CSS risk stratification. (Fig. 10).

## Discussion

Colon cancer is a growing problem in elderly patients owing to its high incidence and the aging of the population [24]. Therefore, late-onset colon cancer should be further emphasized and studied. Faced with limited evidence-based data, oncologists face the challenge of tailoring treatment regimens for patients with late-onset colon adenocarcinoma.

Lymph node status is the most powerful prognostic factor for patients undergoing radical resection for colon cancer, but it is also the most controversial issue [25]. N stage considers only the number of affected lymph nodes and disregards effects related to the total number of lymph nodes examined, which is one of its major drawbacks. In order to explore superior lymph node staging, other parameters, such as negative lymph node count, PLN, LNR and LODDS, have been clinically studied in recent years. Consequently, many new staging systems for lymph nodes have been developed in an attempt to gain a more detailed understanding of a patient's lymph node status by analysing the total number of lymph nodes and the total number of involved lymph nodes.

The LODDS is increasingly favoured by academics as it reduces the reliance on the number of lymph nodes by calculating the ratio of metastatic to non-metastatic lymph nodes.It integrates information on positive and negative lymph nodes to provide a more intuitive picture of tumour burden through a logarithmic ratio. When the number of lymph nodes removed is low or the number of positive lymph nodes is 0, the LODDS provides continuous predictive values, avoiding the LNR, which cannot be calculated when the denominator is 0, and avoiding the problem of discrete grouping in N-staging, thus providing a more continuous and refined risk assessment. Numerous studies on colon cancer have demonstrated that the LODDS has a superior predictive ability for the prognosis of patients in comparison with N, PLN, and LNR staging [10, 26–28].

Our previous research supported this view, so this study was conducted on the basis of the LODDS. Although it has been suggested that LNR is the most accurate lymph node staging system for predicting the prognosis of colon cancer patients undergoing surgical resection, the authors also considered the relatively limited number of patients included in the single-center retrospective study, which may have affected the true results of the comparison of the LNR with the LODDS [29]. Therefore, the development of new prognostic prediction models based on LODDS may lead to more convincing performance.

Our study found that the clinical prognostic predictive ability of the LODDS staging model constructed on the basis of LODDS, T and M stages may be superior to the AJCC 8th edition TNM classification in a Western group of late-onset colon adenocarcinoma patients.

A larger area under the receiver operating characteristic curve (AUC) indicates greater discriminatory power, whereas a lower normalized value of the AIC indicates a better model fit [30]. The results suggest that the LODDS stage model can be considered a better prognostic model for OS and CSS than the other three models because it has the minimum AIC (688080.9 in OS and 296285.2 in CSS), maximum C index (0.713in OS and 0.801 in CSS) and time-dependent AUC. To determine its clinical applicability, we created two nomograms combined with LODDS staging for predicting OS and CSS in patients with late-onset colon adenocarcinoma using data from the training group and then validated the accuracy of the nomograms via data from the test and TCGA groups. The calibration curves revealed a stable linear relationship and accuracy of the nomograms, and the calculated C-index and time-dependent AUC were the highest in both sets. In terms of clinical utility, DCA curves revealed that the net benefit of the nomogram was consistently large, which led us to believe that the nomogram has satisfactory applicability in predicting survival in patients with late-onset colon adenocarcinoma. In conclusion, the LODDS stage model has better predictive accuracy and clinical validity than other staging systems, including the TNM staging system. However, further validation in larger multicenter independent cohorts is needed.

In addition, we risk-graded OS and CSS on the basis of the summed scores of the nomograms and categorized patients with late-onset colon adenocarcinoma into two risk groups. The findings showed that in each cohort, the survival rate was lower in the high-risk group. Notably, the matrix score was greater in the high-risk group than in the low-risk group, which is consistent with previous findings in patients with colon cancer.

Although our study proves that the LODDS stage model has significant advantages in predicting OS and CSS in patients with late-onset colon adenocarcinomas, some of its limitations remain. First, the absence of indicators such as chemotherapy, resection status, and CEA levels may decrease the model's predictive ability. Second, due to the retrospective nature of this study and the small sample size of the TCGA database despite the large sample size of the SEER database, our findings should be validated by prospective multicentre studies with larger sample sizes. Finally, surgical details such as the extent of lymph node dissection at a particular lymph node level may not be well documented and require further study. Despite these limitations, our study successfully demonstrated the very good clinical predictive value of this novel model and, for the first time, included it in the OS and CSS prognostic nomograms of patients with late-onset colon adenocarcinoma.

## Conclusions

This study demonstrates that LODDS may outperform AJCC N staging, PLN, and LNR in predicting the prognosis of late-onset colon adenocarcinoma. Additionally, this study developed a nomogram based on age, gender, race, size, and LODDS stage to predict prognosis, and confirmed its performance. Compared to the 8th edition of the AJCC classification system, our nomogram may exhibit superior survival prediction capabilities, serving as a potential tool to assist in individualized clinical decision-making.

## Supplementary Information

Supplementary file 1

Supplementary file 2

## Data Availability

The relevant data for this study can be obtained from the corresponding author upon reasonable request.
